# Identification and Validation of MYADM as a Novel Prognostic Marker Related to EMT in ESCC

**DOI:** 10.7150/jca.88767

**Published:** 2024-08-19

**Authors:** Qiuxing Yang, Bo Cai, Shudong Zhu, Guomei Tai, Aiguo Shen

**Affiliations:** 1 Cancer Research Center Nantong, Nantong Tumor Hospital, Affiliated Tumor Hospital of Nantong University, Nantong, China.; 2 Nantong Center for Disease Control and Prevention Institute of Chronic Noncommunicable Diseases Prevention and Control, Nantong, China.; 3 Department of Radiotherapy, Nantong Tumor Hospital, Affiliated Tumor Hospital of Nantong University, Nantong, China.; Qiuxing Yang and Bo Cai are co-first authors of this article.; Qiuxing Yang and Aiguo Shen are co-corresponding authors of this article.

**Keywords:** WGCNA, MYADM, ESCC, paclitaxel

## Abstract

**Background:** Esophageal squamous cell carcinoma (ESCC), one of the most aggressive gastrointestinal malignancies, remains an enormous challenge in terms of medical treatment and prognostic improvement. Based on the Gene Expression Omnibus (GEO) and The Cancer Genome Atlas (TCGA) databases in R language, the myeloid-associated differentiation marker (MYADM) was confirmed using bioinformatics analysis and experimental verification. MYADM is upregulated in multiple cancer types; however, the oncogenic mechanism by which MYADM promotes ESCC remains largely unknown.

**Methods:** In the present study, we used weighted gene coexpression network analysis to filter four hub genes (AKAP12, ITGA1, JAM2, and MYADM) in GSE45670 and GSE23400 that are related to the malignant progression of ESCC. Transcription factors and target miRNAs of the hub genes were predicted using the TarBase and JASPRAR databases, respectively, and a regulatory network was established. MYADM was selected based on the analysis of expression differences and prognostic value in ESCC. Next, we confirmed the level of MYADM in ESCC samples using immunohistochemistry of the tissue microarray. The molecular mechanisms of MYADM were further elucidated by experimental analyses, including Transwell assays, wound healing assays, and CCK8.

**Results:** The correlation between MYADM levels and the clinical data of patients with ESCC was confirmed, including tumor differentiation, the node and metastasis stage, T stage, lymphatic metastasis, and postoperative distant metastasis. MYADM was significantly upregulated in ESCC and positively correlated with overall survival. MYADM induced cell proliferation, migration, invasion, and wound healing via the epithelial to mesenchymal transition (EMT) pathway in multiple experiments. Moreover, our results supported the hypothesis that MYADM promotes EMT during paclitaxel resistance.

**Conclusion:** MYADM is closely correlated with ESCC progression, metastasis, and paclitaxel resistance and could be regarded as a novel biomarker and therapeutic target for ESCC patients.

## Introduction

Esophageal cancer (EC) is the seventh and fourth leading cause of cancer-related deaths worldwide and China, respectively 1,2. It has one of the lowest 5-year survival rates among all cancer types: 20% in the United States and 40.1% in China 3. Almost 90% of the patients with EC in China are diagnosed with esophageal squamous cell carcinoma (ESCC), which is the most prevalent type of EC all over the world 4. Unfortunately, its conventional clinical therapeutic effects are still not satisfactory predominantly owing to the poor outcomes of tumor metastasis and recurrence, as well as drug resistance. Tumor proliferation induces tumor angiogenesis, and blood vessels are the main route through which metastatic tumor cells seed distant organs 5,6. Through intervention with angiogenesis inhibitors, the remodeling of the structure and function of abnormal vessels can maximize the efficacy of chemotherapeutic drugs 7. Tumor vasculature provides adequate blood supply for aggressive tumor growth 8. Newly formed tumor blood vessels are typically characterized by significant structural and functional abnormalities, which ultimately contribute to induce tumor epithelial cells to undergo epithelial to mesenchymal transition (EMT) within the tumor microenvironment. As a result, these cells show an increased tendency for migration and metastasis 9. EMT occurs when cancer cells metastasize and promotes cancer infiltration and metastasis by facilitating the ability of cancer cells to move and break down the extracellular matrix 10. Therefore, precise biomarkers targeting the tumor vasculature and EMT are needed for individualized therapy.

The rapid development of high-throughput microarray and sequencing technologies has promoted the derivation of multi-omics public databases such as Gene Expression Omnibus (GEO) and The Cancer Genome Atlas (TCGA) 11,12. An increasing number of researchers are attempting to analyze microarray data and compare cancer atlas with normal atlas to explore the diagnosis, treatment, recurrence monitoring, and drug resistance mechanisms in cancer patients 13. However, public ESCC databases require further investigation to identify the potential biomarkers associated with disease progression. Weighted gene coexpression network analysis (WGCNA) is a bioinformatics tool that can identify hub genes related to ESCC formation, development, and progression 14. In this study, two transcription-level datasets based on the GEO database were analyzed using WGCNA to validate hub genes related to vascular remodeling in patients with ESCC and healthy individuals. Gene Ontology (GO) and Kyoto Encyclopedia of Genes (KEGG) analyses were used to identify biological processes, cellular components, molecular functions, and pathway enrichment based on these hub genes. Subsequently, protein-protein interaction (PPI) network analysis was used to identify all genes in the hub modules as central nodes. Furthermore, the possible upstream and downstream regulatory mechanisms of key vascular remodeling genes were evaluated using large public databases. We also experimentally identified the function of the myeloid-associated differentiation marker (MYADM) in the malignant progression of ESCC.

MYADM was mapped to human chromosome 19q 13.33-q 13.4 by Radiation Hybrid mapping; it consists of three exons and two introns and spans a 7.1-Kb genomic region 15. MYADM encodes a protein with eight putative transmembrane domains and is highly expressed in myeloid cells but is nearly undetectable in cells or tissues of lymphoid origin. MYADM displays tissue expression that is not limited to hematopoietic cells and is also present at significant levels in the brain and lungs. With regard to the hematopoietic system, this novel gene may be useful as a marker of immature hematopoietic cells committed to myelopoiesis 16. The ectopic expression of MYADM was confirmed in non-small-cell lung cancer (NSCLC), acute promyelocytic leukemia, prostate cancer, colon cancer, melanoma, and pancreatic ductal adenocarcinoma 15,17-21. MYADM is involved in the progression of multiple cancers, including proliferation, differentiation, migration, invasion, and tumor immunity 15,17,18. It is also worth noting that MYADM regulates molecular signaling pathways such as the BMP/TGF-β 22, the KLF4/p21 23, and EMT signaling pathways 24. To further elucidate the role of MYADM in ESCC development and progression, we explored its biological role in ESCC.

## Materials and Methods

### Datasets and data preprocessing

All datasets included in this study are available online to the public. In order to find the gene chip data related to ESCC, “esophageal squamous cell carcinoma” was input as the keyword to obtain related data set through the GEO module in the NCBI, and the GSE45670 and GSE23400 microarray data sets were downloaded. The platform for GSE45670 was the GPL570 (HG-U133_Plus_2) Affymetrix Human Genome U133 Plus 2.0 Array, which included 10 adjacent normal tissues and 28 ESCC tissues. The platform for GSE23400 was the GPL97 (HG-U133B) Affymetrix Human Genome U133B Array, which included 51 adjacent normal tissues and 51 ESCC tissues. The platform for GSE75241 was the GPL5175 (HuEx-1_0-st) Affymetrix Human Exon 1.0 ST Array (transcript [gene] version), which includes 15 adjacent normal tissues and 15 ESCC tissues. The platform for GSE26886 was the GPL570 (HG-U133_Plus_2) Affymetrix Human Genome U133 Plus 2.0 Array, which includes 19 adjacent normal tissues and nine ESCC tissues. Transcriptional RNA sequencing data of patients with esophageal carcinoma, including 173 samples, clinicopathological data, and information, were retrieved from TCGA (https://portal.gdc.cancer.gov/). A total of 11236 vascular remodeling related genes (Table S1) were downloaded from GeneCards (https://www.genecards.org/), a searchable and integrative database that automatically integrates gene-centric data from approximately 150 web sources, including genomic, transcriptomic, proteomic, genetic, clinical, and functional information. Eligible patients were histologically confirmed to have thoracic ESCC. Patients with a history of other malignancies were also excluded.

### WGCNA

By constructing a weighted gene coexpression network, the coexpression gene modules were identified, and the association between the gene network and phenotype was explored, as well as the core genes in the network. We calculated the median absolute deviation (MAD) of vascular remodeling related to each gene to eliminate the first 50% of genes with the smallest MAD. Moreover, outliers and samples were removed using the R package of WGCNA's goodSamplesGenes. The soft threshold of GSE45670 was set to 5, and that of GSE23400 was set to 6. The weighted adjacency matrix was transformed into a topological overlap matrix (TOM) to estimate network connectivity, and the hierarchical clustering method was used to construct the cluster tree structure of the TOM matrix. Hierarchical clustering was then performed to identify modules, each of which contained at least 30 genes with a sensitivity of three and a module merging threshold of 0.25. Based on the weighted correlation coefficient of genes, the genes were classified according to their expression patterns. Genes with similar patterns were grouped into a module, and tens of thousands of genes were divided into multiple modules based on gene expression patterns.

### Functional enrichment analysis

A Venn diagram of the overlapping genes was drawn using a dedicated online website (http://bioinformatics.psb.ugent.be/webtools/Venn/). These two datasets were combined to perform GO and KEGG pathway enrichment analyses using the OmicShare tools (https://www.omicshare.com/tools). The Pathway Activity module presents the difference in gene expression between the activation and inhibition groups, which is defined by the pathway scores on the GSCALite website 25. The Ggplot2 and ggridge packages were used to visualize the ridge plots of key genes in the TCGA database. Survival analyses, including the disease-free interval (DFI), disease-specific survival (DSS), overall survival (OS), and progression-free survival (PFS), were performed using the GSCALite website.

### Prediction of key genes‑transcription factors (TFs) ‑miRNAs network

The STRING (https://cn.string-db.org/) online database was used to clarify the interactions between different proteins to construct a PPI network. This study also used Cytoscape 3.8.2 for visual analysis, Pearson's correlation statistical method for the correlation between genes, and the R package corrplot (version 0.84) for the multi-gene correlation map. The Comparative Toxicogenomics Database (CTD, http://ctdbase. org/) 26 assembles interaction data between chemicals, gene products, functional phenotypes, and diseases, affording great convenience for research on disease-associated environmental exposures and potential mechanisms of action of drugs. Using CTD data, the relationship between hub genes and the risk of developing esophageal neoplasms or ESCC was analyzed. To explore the upstream regulators of hub genes, networks were visualized with transcription factor targets derived from the JASPAR TF binding site profile database and comprehensive experimentally validated miRNA-gene interaction data collected from TarBase.

### Correlated genes and post-translational modifications of MYADM in esophageal cancer

LinkedOmics was the first multi-omics database that integrated mass spectrometry-based global proteomics data generated by the Clinical Proteomic Tumor Analysis Consortium on selected TCGA tumor samples 27. PhosphoSite Plus (https://www.phosphosite.org/homeAction) provides comprehensive information and tools for studying protein post-translational modifications including phosphorylation and acetylation. Web access is free for everyone, including commercial users. UbiBrowser (http://ubibrowser.bio-it.cn/ubibrowser_v3/) is an integrated bioinformatics platform for investigating ubiquitin ligase-substrate interaction networks. Moreover, it can systematically analyze the protein network, structure, and sequences involved in the interactions between ubiquitin ligases and substrates. This study predicted the potential protein kinases and interacting E3 ligases in MYADM, which were visualized using Cytoscape.

### Patients and samples

A total of 375 patients who underwent radical esophageal cancer resection between 2010 and 2016 were enrolled. All patients provided detailed clinical data and were followed-up until August 2020. The overall survival period was defined as the time from surgery to death or the last follow-up. Tissue microarray (TMA) specimens included 375 tumor specimens from the Nantong Tumor Hospital and 108 adjacent normal tissues (> 5 cm from the tumor edge). All the patients provided written informed consent on a regular basis. Tumor staging was performed in strict accordance with the International Cancer Alliance standards. This study was approved by the ethics committee of the Nantong Tumor Hospital, and all procedures were performed in accordance with the Guidelines of the World Medical Association Declaration of Helsinki.

### Immunohistochemistry and scoring

Excised esophageal samples were fixed with 10% formalin and embedded in 4-5 mm thick paraffin sections. The tissue chip was deparaffinized, rehydrated, immersed in citric acid buffer (pH 6), and treated with 3% hydrogen peroxide (20 min). The slides were mixed with the primary antibody against MYADM (dilution 1:100, bs-19143R) at 4°C overnight. The secondary antibody was then incubated with the detection kit at 37°C for 20 min. A pathologist observed and scored the immunostained sections of MYADM using an optical microscope. The pathologist was blinded to the patients' clinical data. The staining intensity score ranges of each section were 0 (negative), 1 (weakly positive), 2 (moderately positive), and 3 (strongly positive), and the cell staining ratio was divided into 1 (0-25%), 2 (26 -50%), 3 (51-75%), and 4 (76-100%). The intensity score was multiplied by a proportional score to obtain the final MYADM score: 0-5 is low and 6-12 is high. Kaplan-Meier curves were drawn with the median mRNA values used to divide the tumor samples into high- and low-expression groups using the log-rank test. The tumor and normal groups were compared using Student's t-test.

### Western blot analysis

RIPA lysis buffer (Institute of Biotechnology) was used to extract proteins from the tissue samples and tumor cells. The proteins of interest were separated by 10% SDS-PAGE and transferred to a polyvinylidene fluoride membrane. All membranes were stripped completely. The membrane was blocked by skim milk for 2 h at room temperature, and then incubated with the corresponding primary antibodies, anti-MYADM (diluted 1:1000, bs-19143R), anti-snail antibody (dilution 1:500, ab53319), anti-twist antibody (dilution 1:1000, ab175430), and anti-GAPDH antibody (dilution 1:5000, 60004-2-lg) overnight at 4°C. On the second day, the membrane was incubated with secondary antibodies (diluted mouse secondary antibody 1:5000, rabbit secondary antibody 1:2000, and goat secondary antibody 1:2000) for 2 h at room temperature. Finally, Western Lightning™ chemiluminescence reagent (PerkinElmer, Inc.) was used to visualize the protein, and Tanon High-sig ECL Western blotting Substrate was used for optical density analysis.

### Cell culture and transfection

The human esophageal cancer cell line KYSE150 was purchased from the Cell Bank of the Chinese Academy of Sciences. ECA109 was purchased from Meisen CTCC. All cells were cultured in RPMI 1640 medium (Gibco, California, USA) containing 10% fetal bovine serum (Gibco), 100 U/mL penicillin, and 100 μg/mL streptomycin. All cells were grown in a humidified incubator at 37°C and 5% CO_2_. Interferin siRNA transfection reagent (POLYPLUS, Agent for Building 16, Lane 15, Gudan Road, Pudong New Area, Shanghai) was used for siRNA transfection experiments. The interferin siRNA transfection reagent and plasmid were diluted in RPMI 1640 medium. The MYADM overexpression plasmid (lv-MYADM) was chemically synthesized and packaged into a lentivirus (OBIO Technology Shanghai Corp., Ltd., China), using polybrene (5 μg/mL) to select stably transfected cells. Western blotting was used to verify transfection efficiency.

### Cell counting kit-8 (CCK-8) assay

KYSE150 and ECA109, respectively in lv-MYADM and si-MYADM groups were cultured in a 96-well plate at a rate of 1×100 cells/well, and the culture medium was 100 μL. After inoculation for 24 h, 72 h, 96 h, 10 μL of CCK8 reagent (Dojing Laboratories, High tech Park in Kumamoto Prefecture, Kyushu Island, Japan) was added to each well according to the manufacturer instructions, and incubated at 37°C for 2 h. The optical density (OD) was measured at 450 nm using a microplate reader (Thermo Scientific, Waltham, Massachusetts, USA). To study paclitaxel sensitivity, the cells were seeded in 96-well plates overnight at a density of 5×10^4^ cells/well. After the cells adhered, 0, 2.5, 5, 10, 20, 40, or 80 ng/mL paclitaxel was added. After 48 h of treatment, 10 μL of CCK8 reagent was added to each well, and plates were incubated at 37°C for 2 h, and then absorbance was measured at 450 nm. Cell viability was calculated as: (experimental group OD value blank group OD value) / (control group OD value-blank group OD value) × 100%.

### Migration and invasion assay

After the cells were transfected with siRNA or overexpressed lentivirus, they were digested with trypsin (ethylenediaminetetraacetic acid trypsin), and then esophageal cancer cells were suspended in gepe (serum-free medium). In the migration experiment, esophageal cancer cells (1×10^5^ cells/well) were resuspended in basal cells and seeded in the upper chamber (200 μL) of a 24-well transwell (Costar #3422, 2 Alfred Rd, Kennebunk ME 04043 USA) plate. The lower chamber contains 500 μL RPMI 1640 medium (containing 10% FBS Serum-free medium (100 μL) containing Matrigel (Corning, Discorery Labware, Inc. Two Oak Park Bedlord, MA 01730 USA) to the upper chamber of a 24-well Transwell plate for invasion experiments. The dilution ratio of matrigel was 1:9. The number of cells used in invasion experiments was 2×10^5^ cells/well. The migration experiment was incubated for 24 h, and invasion experiments were incubated for 48 h. The cells were then fixed with 4% paraformaldehyde for 20 min in the lower chamber and stained with crystal violet for 5 min. The cells on the lower periventricular membrane were counted under an optical microscope (200× magnification). Five areas were randomly selected for cell counting. The experiment was repeated three times and similar results were obtained. Data represent mean ± SEM and the *P*-values were determined by Chi-square test.

### Wound healing assay

Cells transfected with siRNA or overexpression lentivirus were inoculated into 6-well plates and cultured in RPMI-1640 supplemented with 10% FBS until the cell density reached > 90%. Use a 100 μL pipette to scratch the cell monolayer along a straight edge perpendicular to the horizontal line, and incubate with a serum-free medium. The serum-free medium was changed every 0, 24, and 48 h and the cells were photographed (magnification 100x) with a LEICA DMi8 inverted microscope. Data represent mean ± SEM, n = 3 independent repeats. *P*-values were determined using the chi-square test.

### Statistical analysis

All data were calculated based on the average standard deviation. SPSS 26.0, GraphPad Prism 7.0, and ImageJ software were used for statistical analysis. The χ^2^ test was used to evaluate the correlation between the expression of MYADM in clinicopathological parameters. The Cox proportional hazards regression model was used for the univariate and multivariate analyses of prognosis. The Kaplan-Meier method and log-rank test were used to generate survival curves. Comparisons between the experimental and control groups were performed using the t-test. *P* < 0.05 was considered statistically significant.

## Results

### Identification of hub VRmRNAs

**Figure 1:**
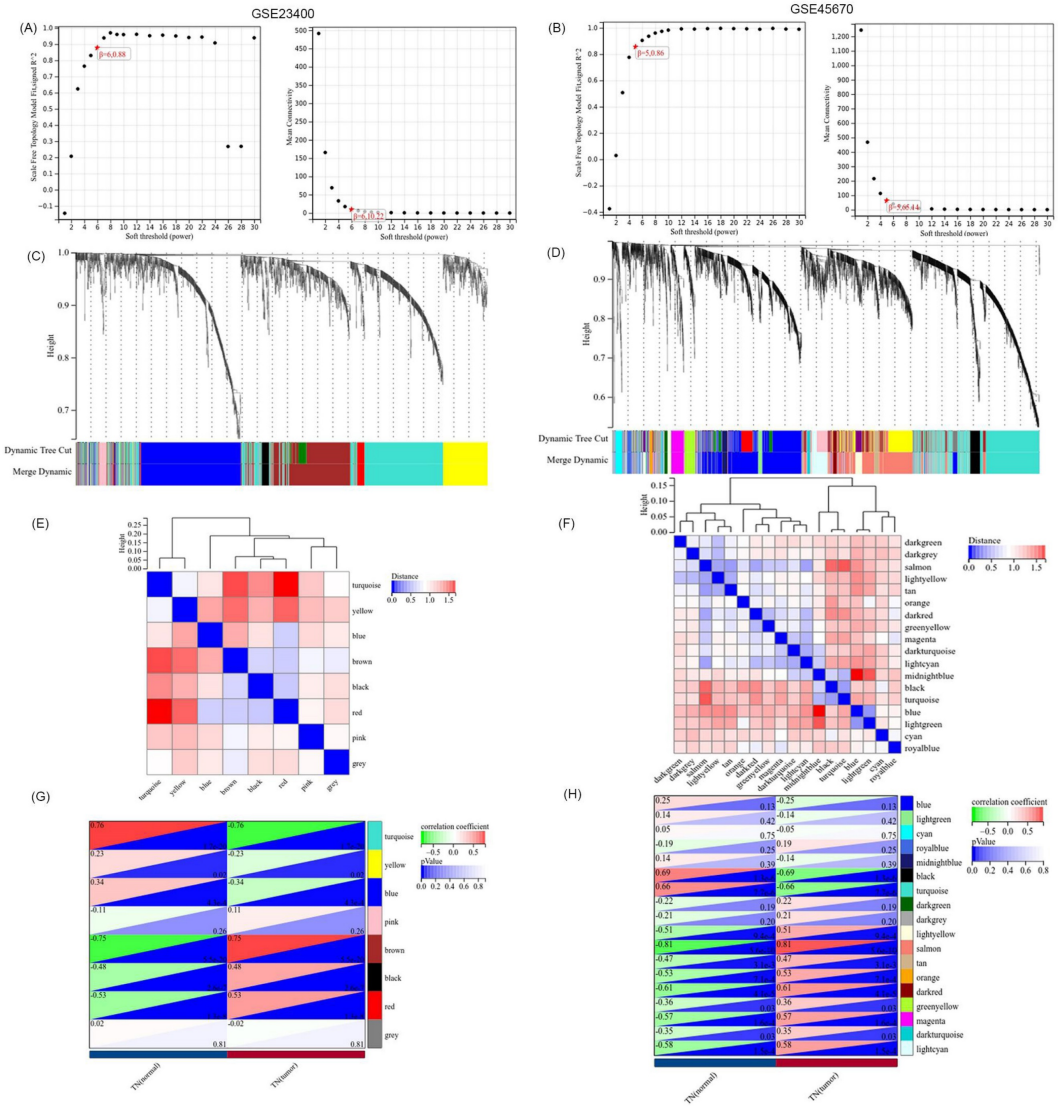
** Identification of VRmRNAs by weighted gene coexpression network analysis (WGCNA).** The scale-free fit index and the average connectivity of soft threshold power are confirmed for GSE23400 (A) and GSE45670 (B). (C,D) Dendrogram of all VRmRNAs clustered based on the measurement of dissimilarity. The color band shows the results obtained from the automatic single-block analysis. (E,F) Module eigenvector clustering. The correlation of these modules between the normal group and tumor group for GSE23400 (G) and GSE45670 (H).

WGCNA was performed to identify modules of highly correlated genes. It also summarizes the interconnections between the modules and their associations with external sample traits to identify candidate biomarkers or therapeutic targets 28. First, 11236 vascular remodeling related mRNAs (VRmRNAs) were obtained from GeneCards using the keyword “vascular remodeling” (Table S1). Subsequently, we extracted the RNA profiles of two datasets: GSE23400 (51 ESCC samples and 51 normal esophageal epithelial samples) and GSE45670 (28 ESCC samples from patients who received neoadjuvant chemoradiotherapy and 10 normal esophageal epithelial samples). In WGCNA, selected β = 6 for GSE23400 and β = 5 for GSE45670 to construct a scale-free network (Figure 1A and 1B). As shown in Figure 1C and 1D), different module genes in the dynamic tree cut were reclustered through a topological similarity strategy, with the number of genes per module not less than 30, and sensitivity = 3. For the GPL97 platform in GSE23400, eight coexpression modules were clustered, with the turquoise module (Cor = 0.76, *P* = 1.7×10^-20^), and blue module (Cor = 0.34, *P* = 4.3×10^-4^) as the top 2 strongest positive correlation with ESCC (Figure 1E, 1G). For GSE45670, 18 coexpression modules were clustered, with the black module (Cor = 0.69, *P* = 1.3×10^-6^), and turquoise module (Cor = 0.66, *P* = 7.7×10^-6^) having the top 2 strongest positive correlations with ESCC (Figure 1F and 1H). Based on the cutoff criteria (|MM| > 0.8, |GS| > 0.1), VRmRNAs with high connectivity in the clinically significant modules were identified as hub genes.

### PPI network analysis and enrichment analysis of VRmRNAs

**Figure 2:**
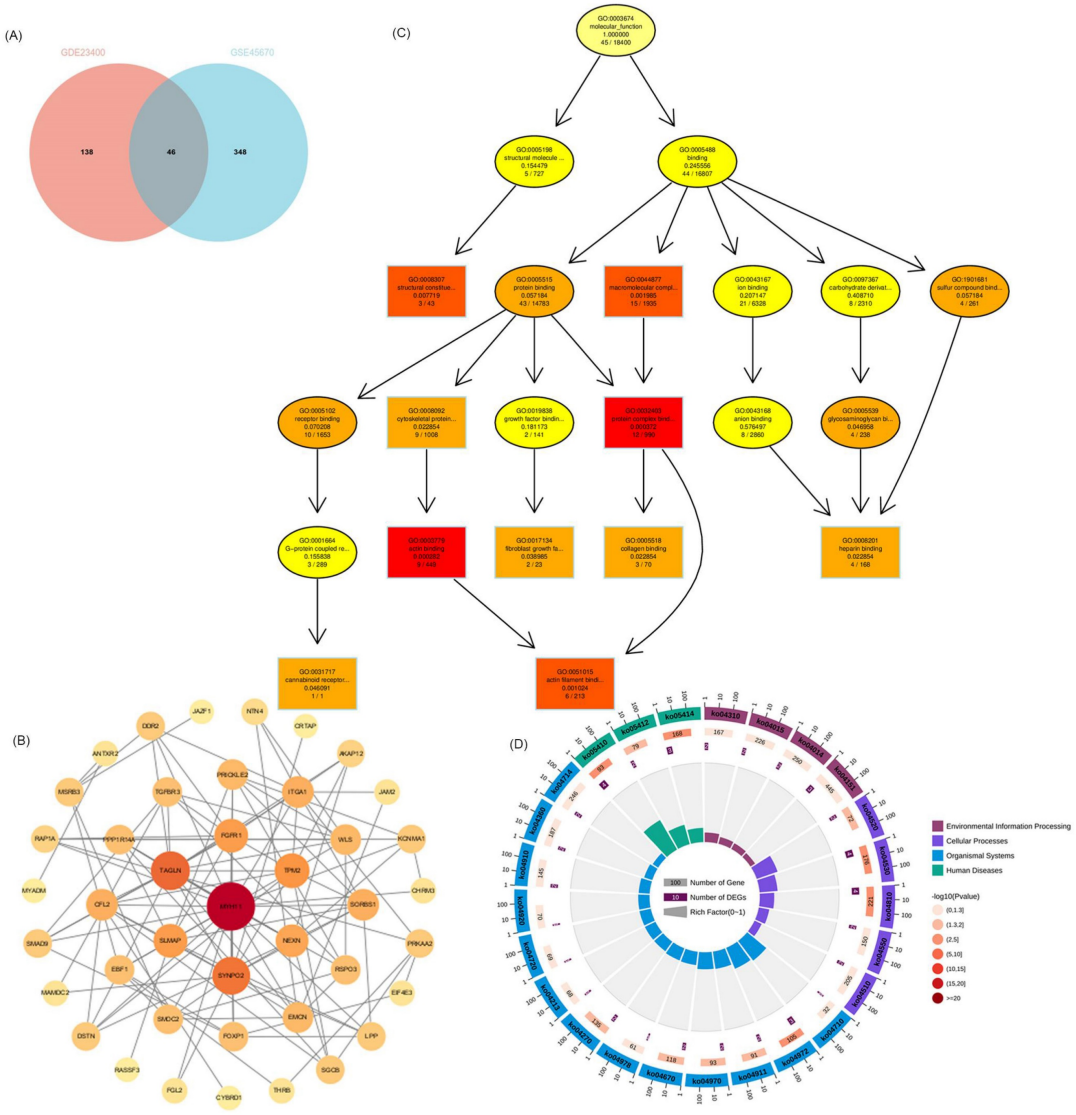
** Functional analyses and construction of the VRmRNAs.** (A) The venn diagram of VRmRNAs in top2 modules. (B) The PPI network of 46 eligle genes. (C) GO enrichment analysis. (D) KEGG enrichment analysis. GO, Gene Ontology; KEGG, Kyoto Encyclopedia of Genes and Genomes; LASSO, Least Absolute Shrinkage and Selection Operator.

GO and KEGG were used to identify and confirm related biological processes. We used Venn analysis with the data of hub gene sets based on the GSE23400 and GSE45670 datasets to further validate the reliability of VRmRNAs in ESCC (Figure 2A). As shown in Figure 2B, Cytoscape software was utilized for PPI network analysis to determine the interaction among 46 VRmRNAs (Table S2). These genes were screened using GO and Kyoto Encyclopedia of Genes and Genomes enrichment analyses. GO analysis indicated that these genes were enriched for actin binding, protein complex binding, actin filament binding, macromolecular complex binding, structural constituents of muscles, cytoskeletal protein binding, collagen binding, heparin binding, and glycosaminoglycan binding (Figure 2C). KEGG analysis showed that hypertrophic cardiomyopathy, tight junctions, regulation of the actin cytoskeleton, dilated cardiomyopathy, adherens junctions, and leukocyte transendothelial migration were enriched in these key genes (Figure 2D).

### Relationship between hub 4 VRmRNAs and pathway activity

**Figure 3:**
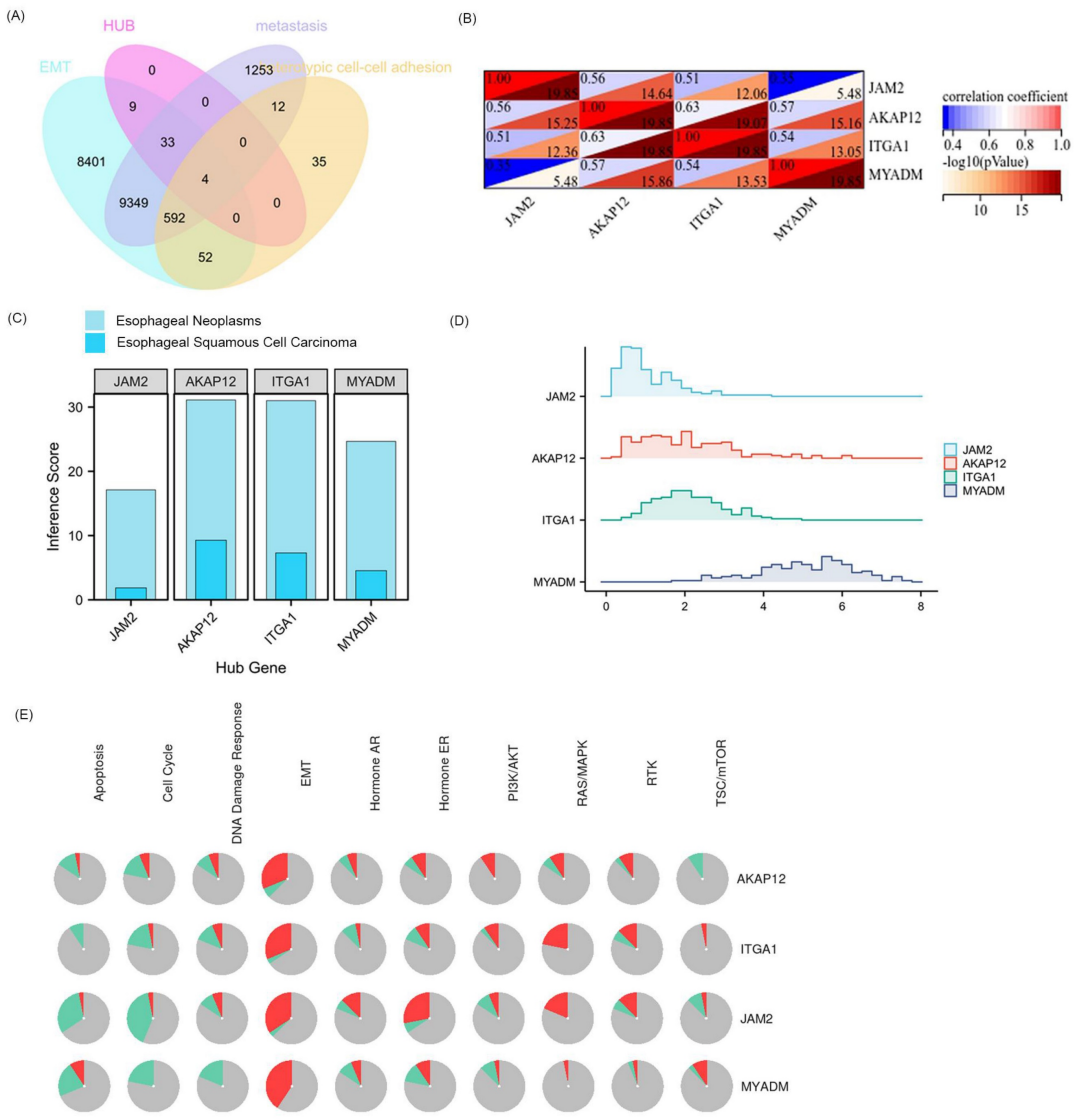
** Extraction and analysis of hub EMT-related genes in ESCC.** (A) The venn diagram of module genes, EMT, metastasis, and heterotypic cell-cell adhesion-related genes. (B) The heatmap of the relationship between these 4 key genes. (C) 4 key genes related to esophageal neoplasms and esophageal squamous cell carcinoma diseases based on the CTD database. (D) The ridge plot displayed the expression of 4 key genes in TCGA. (E) Pathway activity analysis of the individual four genes in ESCA.

Public CTD is an innovative digital ecosystem that connects toxicological information on chemicals, genes, phenotypes, diseases, and exposures to advance our understanding of human health 26. According to the above enrichment analysis, VRmRNAs were associated with cell adhesion and tumor metastasis. As is well-known, EMT plays an important role in tumor initiation, invasion, metastasis, and resistance to therapy 29. Therefore, we collected the overlapping points of VRmRNAs, EMT, metastasis, and heterotypic cell-cell adhesion-related genes to obtain four key genes: AKAP12, ITGA1, JAM2, and MYADM (Figure 3A). Figure 3B shows a heat map of the relationship between these four key genes. As predicted by applying the CTD database, AKAP12 showed the highest association with esophageal neoplasm/esophageal squamous cell carcinoma, whereas JAM2 showed the lowest correlation (Figure 3C). The ridge plot in Figure 3D indicates that JAM2, and AKAP12, ITGA1expression was relatively concentrated in 1 and 2, while MYADM expression was relatively concentrated in 6 in the TCGA database. A pie chart (Figure 3E) was constructed to assess the possible mechanisms involving these genes. The results showed that all four genes were related to the EMT and RAS/MAPK pathways, and could regulate the TSC/mTOR pathway, indicating their critical role in cancer.

### Hub VRmRNAs‑TFs‑miRNAs regulatory network

**Figure 4:**
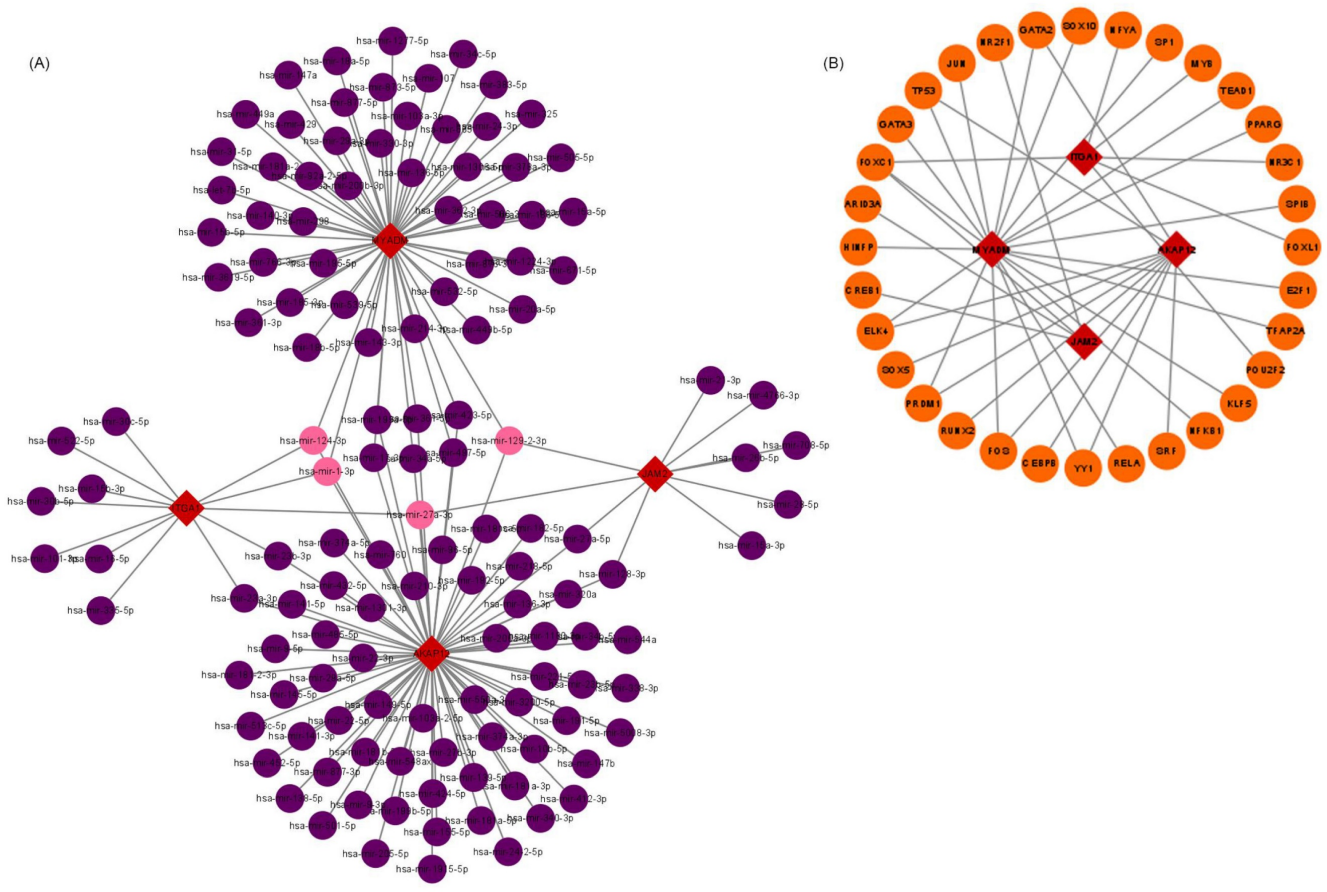
** Relationship between hub 4 genes-TFs-miRNAs regulatory network. (**A) miRNA-hub 4 genes regulatory network: the red squares represent hub 4 genes, the purple dots represent miRNA, and the pink dots represent 4 key miRNA. (B) TF-hub 4 genes regulatory network: the red squares represent hub 4 genes, and the yellow dots represent transcription factors.

The upstream regulation of hub VRmRNAs was explored by predicting related TFs and miRNAs. miRNAs of hub VRmRNAs were predicted with TarBase, and a hub VRmRNA-miRNA regulatory network that involved 149 nodes was generated (Figure 4A). Four miRNAs with interactions were identified: miR-129-2-3p interacted with AKAP12, JAM2, and MYADM; miR-27a-3p interacted with AKAP12, ITGA1, and JAM2; and miR-1-3p and miR-124-3p interacted with AKAP12, ITGA1, and MYADM. The TFs of hub VRmRNAs were predicted using JASPRAR, and a hub VRmRNA-TF regulatory network comprising 40 TFs was constructed (Figure 4B). There were six TFs with interactions: FOXC1 interacted with ITGA1, JAM2, and MYADM; and ELK4, PRDM1, FOS, YY1 and GATA2 interacted with AKAP12 and MYADM. Figure S1 shows the relationship between the hub 4 genes and the TF-miRNA regulatory network. However, further validation is required in this regard.

### Validation of core gene MYADM

**Figure 5:**
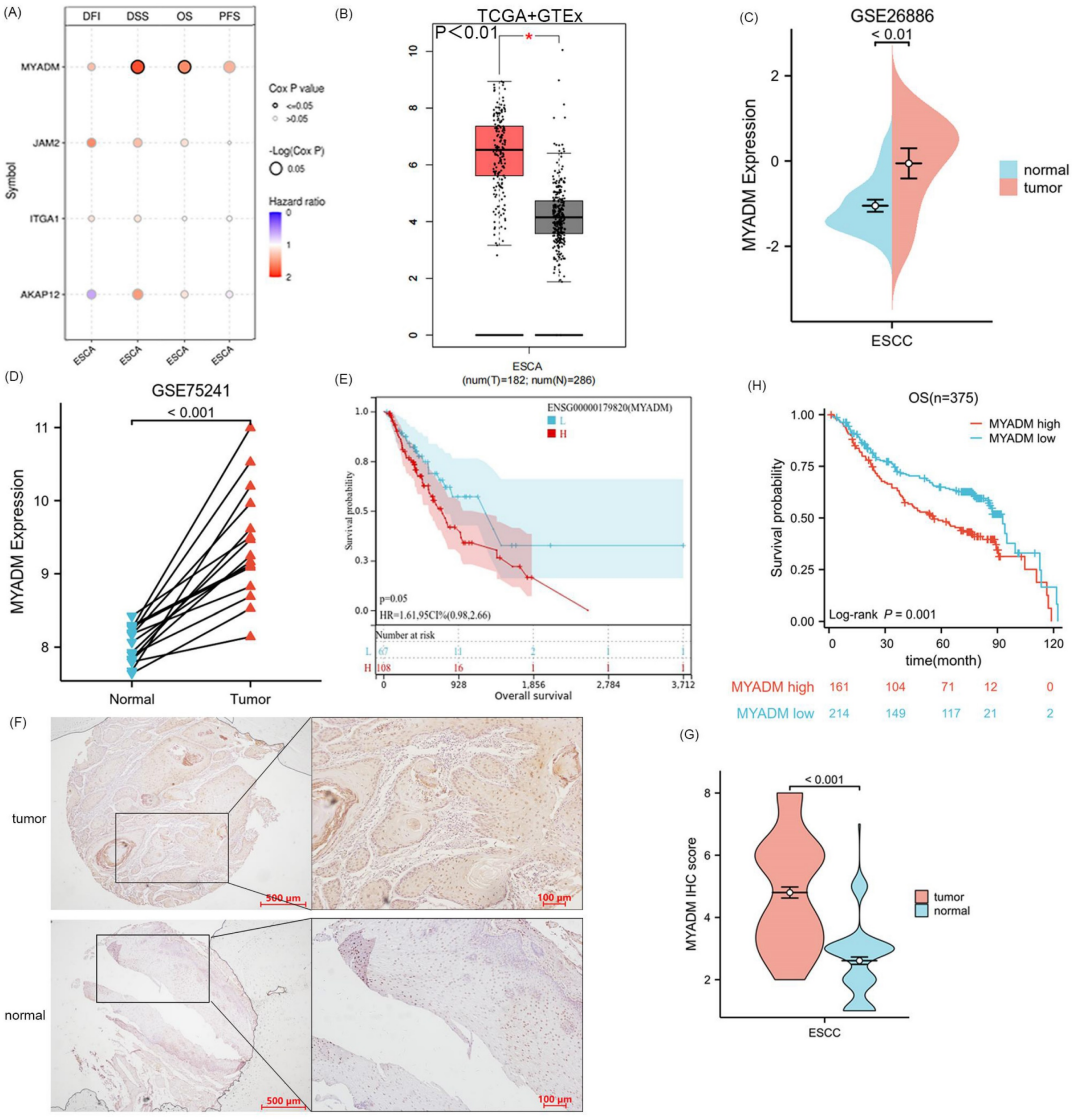
** Analysis and validation of core gene MYADM.** (A) The survival analysis of four key genes in ESCA, including DFI, DSS, OS, and PFS. Identification of the mRNA level of MYADM based on the TCGA (B) database, GEO database GSE26886 (C) and GSE75241 (D) in ESCC patients. (E) The OS analysis of MYADM based on TCGA. (F, G) The protein expression level of MYADM in normal esophageal tissue samples and ESCC tissue samples. (H) The OS analysis of MYADM based on 375 tissue microarray (TMA).

Since the four genes AKAP12, ITGA1, JAM2, and MYADM were defined as the core genes associated with ESCC, we performed a survival analysis of the four key genes in ESCA, including DFI, DSS, OS, and PFS. Remarkably, only OS (*P* = 0.042) and DSS (*P* = 0.028) of the MYADM group were significantly different (Figure 5A). Additionally, MYADM showed the largest difference in expression between these tumor tissues and the corresponding normal tissues in the Genotype-Tissue Expression (GTEx) database (Log_2_FC > 2, *P* < 0.01, Figure 5B). Furthermore, we used the GSE26886 (*P* < 0.01, Figure 5C) and GSE75241 (*P* < 0.001, Figure 5D) databases to confirm MYADM expression, which indicated that MYADM was enhanced in ESCC. The effect of MYADM on ESCA prognosis was further evaluated using TCGA, and patients with high MYADM expression had a poorer prognosis (*P* = 0.05, Figure 5E). The reason for the statistical insignificance may be the limitations of the sample size. Furthermore, we used immunohistochemistry (IHC) staining to detect these proteins in ESCC and the corresponding paracancerous esophageal tissues. As predicted, MYADM expression was higher in ESCC tissues than in the corresponding paracancerous esophageal tissues (*P* < 0.001, Figure 5F, 5G). In the survival analysis, we found that in ESCC, high expression of MYADM was associated with a worse survival rate, and low expression of MYADM was associated with a higher survival rate (*P* = 0.001, Figure 5H). Furthermore, we found that MYADM was closely correlated with some clinical parameters in 375 ESCC TMA. The results of the chi-square test indicated that high MYADM expression was significantly associated with tumor differentiation (*P* = 0.043), the node and metastasis (TNM) stage (*P* = 0.021), T stage (*P* = 0.036), lymphatic metastasis (*P* = 0.030), and postoperative distant metastasis (*P* = 0.003), but not with age, sex, vascular invasion, or nerve infiltration (Table 1). To determine whether the expression level of MYADM was an independent prognostic factor for patients with ESCC, univariate and multivariate analyses were performed. This result may validate the fact that the T stage (*P* = 0.031), TNM stage (*P* = 0.036), and MYADM stage (*P* = 0.036) were independent prognostic factors for ESCC (Table 2). These results indicate that MYADM may play a key role in the development and progression of ESCC.

### Kinases correlated with MYADM in ESCC

**Figure 6:**
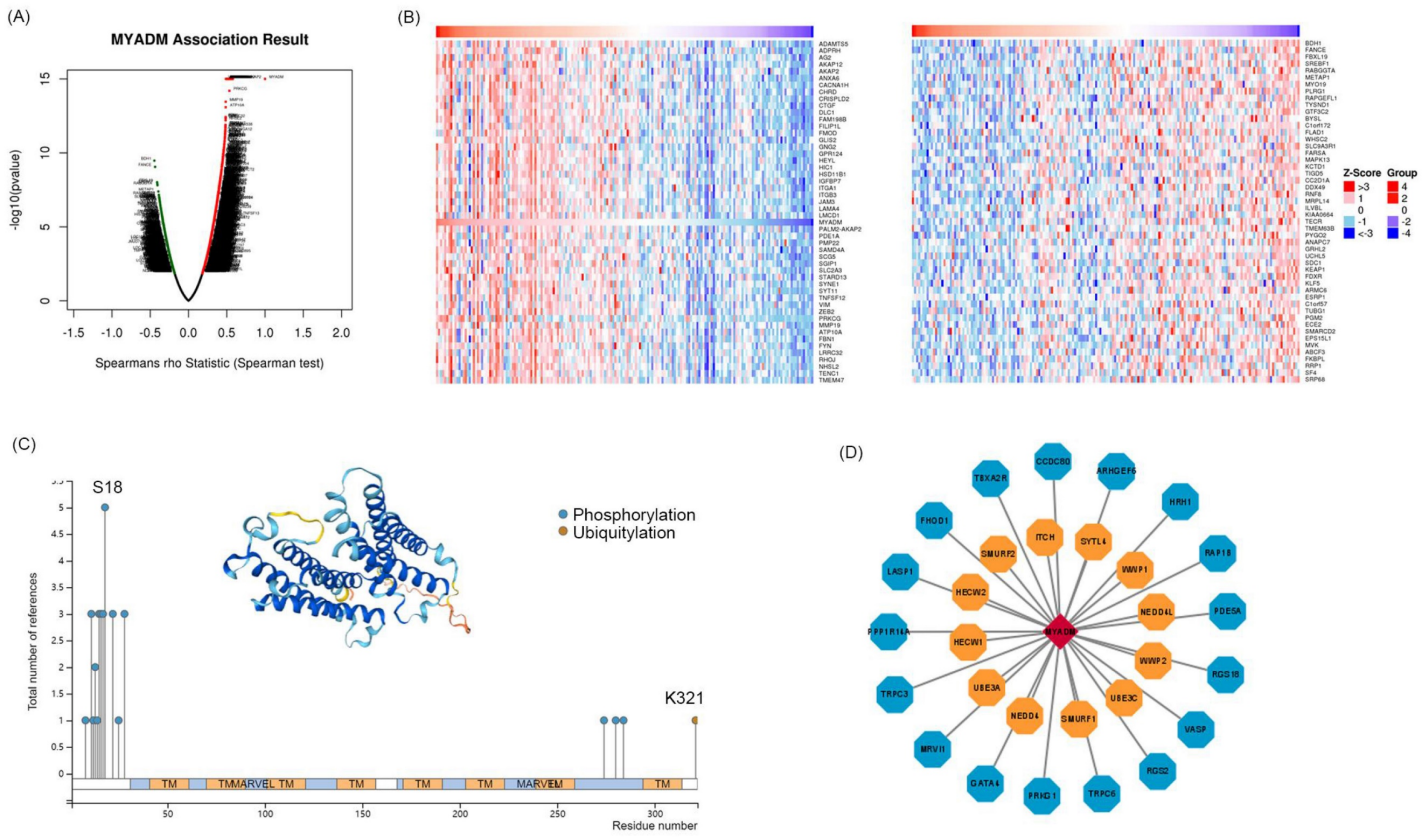
** Kinases associated with MYADM expression in ESCC.** (A) Correlations between MYADM and differently expressed genes from LinkedOmics database. (B) Heat maps show the top 50 genes that are positively or negatively correlated with MYADM. (C) Summary of the MYADM phosphorylation and ubiquitylation sites via PTM dataset. (D) protein kinases and E3 ligases related to MYADM.

To identify the core kinases of MYADM in ESCC, we used the LinkedOmics database. The volcanic map in Figure 6A shows the genes related to MYADM. According to the Spearman test, the top 50 positively and negatively correlated genes were identified and analyzed using heat maps (Figure 6B). Moreover, we summarized MYADM phosphorylation and ubiquitylation in the PhosphoSitePlus database V6.7.1.1. Figure 6C provides a brief description of MYADM, and the most significant sites are the phosphorylation sites of S18. The ubiquitylation site was K321 in MYADM. To identify the potential translational modification kinases of MYADM, the UbiBrowser web tool was used to query the ubiquitin ligase (E3) that targeted MYADM. Seventeen protein kinases were related to MYADM, with ARHGEF6 having the highest score (7.4158) (Figure 6D). Twelve E3 ligases were predicted to target MYADM, with NEDD4 showing the highest score of 0.896.

### MYADM promoted the process of EMT in vitro

**Figure 7:**
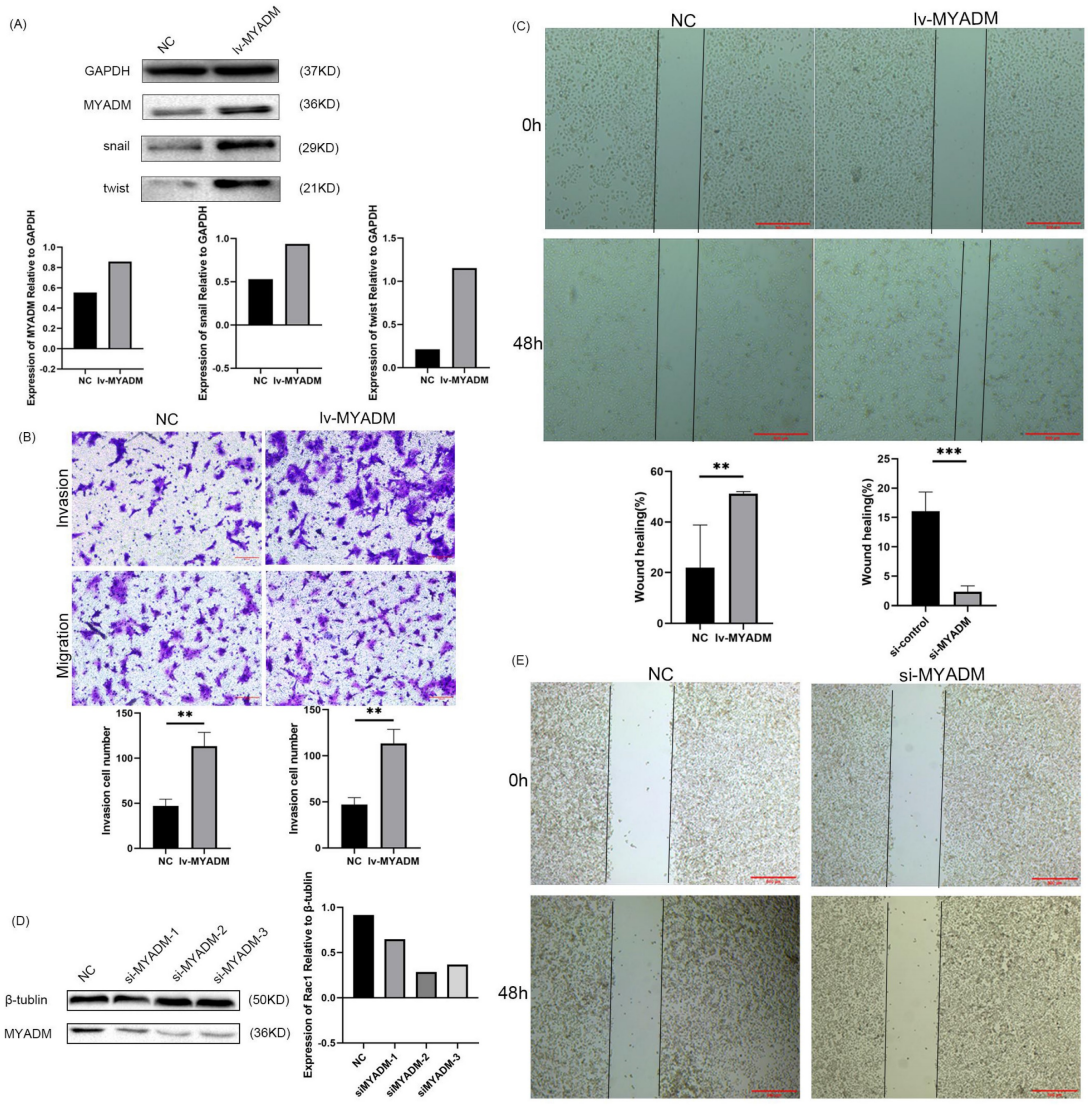
** MYADM regulated EMT to promote the metastasis of ESCC.** (A) Western blot analysis of MYADM and EMT protein expression in NC and lv-MYADM groups. (B) The cell invasion and migration were significantly increased after overexpressing MYADM in ESCC cell line. (C) The wound healing assay was also carried out. (D) Immunoblot analysis of MYADM protein at 48h posttransfection of siR-NC and siR-MYADM in ECA109. β-tublin was used as an internal reference. (E) The wound healing assay was performed to test migration of wound healing ability by applying Image J and Graphpad Prism 8. Scale bar: 500μm. Data represent mean±SEM, n = 3 independent repeats. The *P*-values were determined by Chi-square test. *P<0.05*,* **P<0.01*,* ***P<0.001*.*

Owing to the overexpression of MYADM in ESCC, we speculated that MYADM might function as an oncoprotein. To test this hypothesis, we first overexpressed the lentivirus in KYSE150 cells (Figure 7A) and then knocked down MYADM expression in ECA109 cells using siRNAs (Figure 7D). The clinical parameters in the TMA indicated a link between MYADM expression and lymph node metastasis. Therefore, we used Transwell and wound healing assays to investigate the effects of MYADM expression on the migration and invasion of ESCC cells. MYADM overexpression significantly increased cell invasion and migration (Figure 7B). The results of the wound healing experiments suggested that the overexpression of MYADM significantly promoted the migration of ESCC cells compared to that of control cells (Figure 7C). Conversely, these abilities significantly decreased after MYADM knockdown (Figure 7E). As shown in Figure 7A, western blot analysis was performed to detect the expression of Snail and Twist. The increase in Snail and Twist levels verified that MYADM overexpression markedly promoted EMT.

### MYADM enhanced paclitaxel resistance

**Figure 8:**
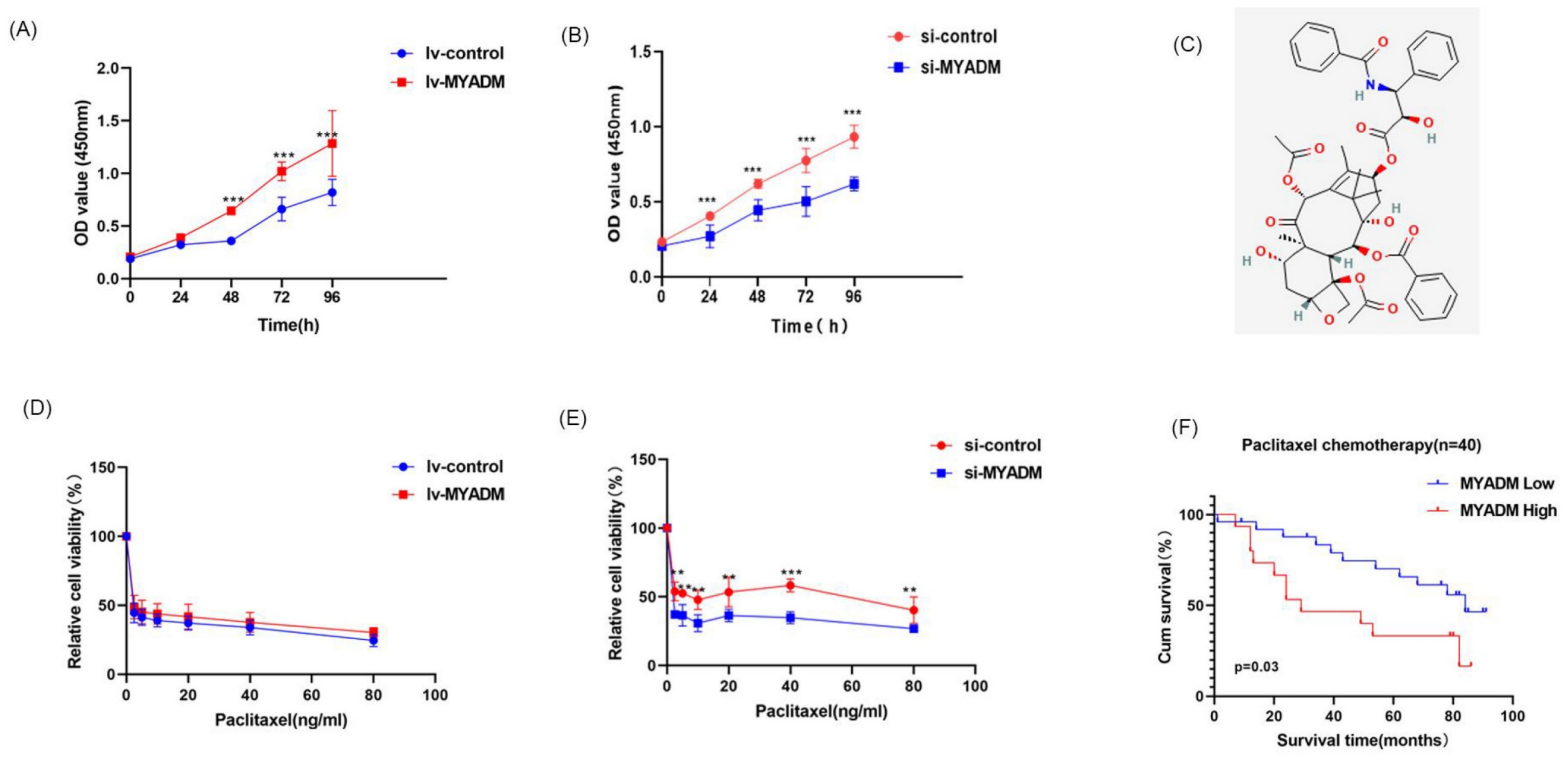
** MYADM enhanced paclitaxel resistance.** CCK8 assay was performed to assess proliferation in ESCC cell at a specific time, respectively in lv-MYADM (A) and si-MYADM (B) groups. (C) The structure of paclitaxel in PubChem. The cell viability was examined by CCK8 assay of KYSE150 treated with paclitaxel for 48h in lv-MYADM (D) and si-MYADM (E) groups. (F) OS based on the MYADM expression in 40 patients with paclitaxel chemotherapy.

The CCK8 experiment was used to verify the proliferative effect of MYADM in ESCC. Compared with the control group, the proliferation rate of ESCC cells was enhanced in the lv-MYADM group (Figure 8A). In contrast, MYADM knockdown completely inhibited ESCC cell proliferation (Figure 8B). Therefore, we conclude that MYADM promotes ESCC proliferation. Paclitaxel (Figure 8C) is a promising chemotherapeutic drug for esophageal cancer, with an efficacy of approximately 32% as a monotherapy in patients with locally advanced and metastatic esophageal cancer 30. Therefore, we investigated whether MYADM was related to the chemical sensitivity of ESCC cells to paclitaxel. ESCC cells were treated with different concentrations of paclitaxel, and the CCK-8 assay was performed to measure cell viability. These results indicate that lv-MYADM significantly reduced the sensitivity of paclitaxel to PTX (Figure 8D). Moreover, MYADM knockdown enhanced the sensitivity of ESCC cells to paclitaxel and reduced their survival rate (Figure 8E). According to the in vivo analysis of 40 patients who received paclitaxel chemotherapy after surgery, patients with high MYADM expression had a worse prognosis, and patients with low MYADM expression had a better prognosis (*P* = 0.03, Figure 8F). Therefore, we concluded that MYADM inhibited the chemotherapeutic sensitivity of paclitaxel in ESCC through EMT.

## Discussion

ESCC is one of the most aggressive gastrointestinal malignancies and surgery is the main curative treatment for resectable ESCC 31. However, the overall prognosis, with a 5-year overall survival rate of 15-25%, remains poor 32. According to the American Joint Committee on Cancer guidelines, TNM classification system is widely used to evaluate ESCC prognosis worldwide 33. Esophageal carcinoma cells directly infiltrate and spread through blood or lymphatic vessels and nerves, which is a precursor of tumor metastasis and disease progression, with a pattern of manifestation of invasive characteristics 34,35. Cancer progression occurs concomitantly with profound remodeling of the cellular microenvironment. Tumor endothelial cells (TEC) lining the tumor blood vessels energetically contribute to tumor progression and metastasis. In addition to tumor cells, TEC may develop drug resistance during clinical tumor treatment, allowing malignant tumor cells to survive chemotherapy and metastasize 36. The development of drug resistance is accompanied by remodeling of vascular morphology, which is potentially manipulated by tumor-secreted proangiogenic factors 37. Intervention with angiogenesis inhibitors can remodel the structure and function of abnormal vessels to maximize the efficacy of chemotherapeutic drugs 7. Anti-angiogenic therapies also have side effects such as deterioration of the tumor microenvironment, leading to tumor progression and increased EMT. Thus, revealing the molecular mechanisms of ESCC tumorigenesis and metastasis may help identify novel biomarkers and potential drug targets for ESCC patients.

In our study, we found that four genes (AKAP12, ITGA1, JAM2, and MYADM) were closely correlated with the formation, development, and progression of ESCC using the WGCNA network based on the GSE23400 and GSE45670 datasets. Deacetylation of AKAP12 at K531 by HDAC6 increases its ubiquitination, which facilitates AKAP12 proteasome-dependent degradation to promote colon cancer metastasis 38. Hsa_circ_0110757 inhibits glioma cell apoptosis by sponging hsa-miR-1298-5p to promote ITGA1 expression, which could be a potential therapeutic target for reversing glioma resistance to temozolomide 39. Overexpression of JAM2 blocks the invasion and migration of breast cancer cells by inhibiting the EMT pathway 40. We used the TCGA database to verify and further analyze the correlation between the four hub genes and prognosis, which indicated that MYADM played a key role in the carcinogenesis, metastasis, and paclitaxel resistance of ESCC. The expression of MYADM, a novel hematopoiesis-associated marker composed of 322 amino acids, was increased in the neointima, suggesting that MYADM is a potential target for vascular remodeling 41. Previous studies identified a new regulatory model of the miR-182-3p/MYADM/KLF4/p21 axis in pulmonary hypertension vascular remodeling 23. Moreover, a recent study reported high levels of MYADM in multiple cancer types. MYADM is strongly associated with OS and DFS in patients with NSCLC, and high MYADM expression is associated with a poor prognosis 17. Researchers integrated scRNA-seq and bulk sequencing data to identify biomarkers that affect pancreatic ductal adenocarcinoma survival and identified MYADM as one of the differentially expressed genes in ductal cells between normal and tumor tissues 21. We found that MYADM expression was increased in ESCC tissue samples and was positively associated with OS in patients with ESCC. Additionally, MYADM was highly expressed in patients with ESCC in two independent datasets. These results indicated that MYADM, as an oncogene, plays a key role in multiple cancers and may be an effective biomarker for patients with cancer, especially ESCC. However, the mechanisms underlying MYADM overexpression remain unclear. In our opinion, the upregulation of MYADM may be due to phosphorylation, ubiquitination (Figure 6C), microRNA regulation, or activity of key transcription factors (Figure 4). The prediction of transcription factors and miRNAs was externally validated using another database to improve credibility in Figure S1. miRNAs of hub VRmRNAs were predicted using miRDB, and a hub VRmRNA-miRNA regulatory network involving 432 nodes was generated (Figure S1A). Three of the four key miRNAs shown in Figure 4A were included. miR-124-3p interacted with MYADM and JAM2. In contrast, miR-1-3p and miR-129-2-3p interacted with AKAP12. The TFs of hub VRmRNAs were validated using ENCODE, and a hub VRmRNA-TF regulatory network comprising 65 TFs was constructed (Figure S1B). However, based on ENCODE, SOX5 was predicted to interact with ITGA1 instead of AKAP12. SP1 interacted with MYADM in both databases. Based on these findings, the transcription factor SP1 likely regulates MYADM expression. However, this hypothesis requires further validation.

Moreover, we found that MYADM expression was significantly correlated with multiple clinicopathological parameters based on IHC staining, including tumor differentiation, TNM stage, T stage, lymphatic metastasis, and postoperative distant metastasis. This is the first study to show that MYADM was significantly enhanced in ESCC samples compared to normal esophageal samples and that MYADM was an independent prognostic marker for ESCC patients. In addition, MYADM was positively associated with macrophages, neutrophils, and dendritic cells in both lung squamous cell carcinoma and lung adenocarcinoma (Cor > 0.3, *P* < 0.05), suggesting its role in regulating tumor immunity 17. Based on the research content of the present study and previously published literature, the combination of chemotherapy and immunotherapy has translational potential for clinical applications in ESCC treatment.

A previous study showed that the expression of the main regulators of EMT, invasion, motility, and migration changed after MYADM knockdown, suggesting that overexpressed MYADM probably predicted subsequent lymph node metastasis in patients with prostate cancer 24. Therefore, we further explored the downstream regulation of MYADM expression. In the present study, we first confirmed that MYADM overexpression promoted the proliferation, migration, and invasion of ESCC cells. Figure 7 shows that MYADM overexpression not only contributed to an increase in migration and invasion, but also promoted wound healing in ESCC. Thus, MYADM silencing effectively suppressed this phenomenon. Taken together, these results indicate that MYADM promotes metastasis and EMT in ESCC cells.

Moreover, this is the first study to verify that MYADM affects paclitaxel sensitivity via the EMT in ESCC. According to tissue microarray analysis, high MYADM levels were strongly associated with poorer OS in ESCC patients treated with paclitaxel. To confirm this, a CCK8 assay was performed to study the effect of MYADM on paclitaxel sensitivity. The results showed that enhanced MYADM expression increased the survival rate of ESCC cells and led to paclitaxel resistance. Thus, targeting MYADM may enhance the clinical chemotherapeutic efficacy of paclitaxel and may suppress the progression and metastasis of ESCC.

To the best of our knowledge, this is the first study to systematically elucidate the expression, function, and molecular mechanisms of MYADM during ESCC progression. We demonstrated that MYADM increased proliferation, migration, invasion, metastasis, and paclitaxel resistance by regulating EMT. In summary, this study further confirmed that MYADM could be a diagnostic marker and potential therapeutic target for patients with ESCC.

## Supplementary Material

Supplementary figures and tables.

## Figures and Tables

**Table 1:** Correlation analysis of MYADM expression and clinicopathological characteristics in 375 cases of ESCC.

Clinicopathological features		Total	MYADM		
			Negative (n=232)	Positive (n=143)	*P* value
**Age**	≤65	200	123 (61.5%)	77 (38.5%)	0.876
	>65	175	109 (62.3%)	66 (37.7%)	
**Gender**	female	95	59 (62.1%)	36 (37.9%)	0.956
	male	280	173 (61.8%)	107 (38.2%)	
**Differentiation**	Well	105	74 (70.5%)	31 (29.5%)	0.043*
	Moderate	149	82 (55.0%)	67 (45.0%)	
	Poor	121	76 (62.8%)	45 (37.2%)	
**pTNM stage**	I-II	217	145 (66.8%)	72 (33.2%)	0.021*
	III-IV	158	87 (55.1%)	71 (44.9%)	
**T stage**	≤T2	154	105 (68.2%)	49 (31.8%)	0.036*
	>T2	221	127 (62.0%)	94 (38.0%)	
**Lymphatic metastasis**	Yes	170	95 (55.9%)	75 (44.1%)	0.030*
	No	205	137 (66.8%)	68 (33.2%)	
**Vascular invasion**	Yes	40	23 (57.5%)	17 (42.5%)	0.547
	No	335	209 (62.4%)	126 (37.6%)	
**Nerve invasion**	Yes	55	28 (50.9%)	27 (49.1%)	0.070
	No	320	204 (63.8%)	116 (36.2%)	
**Distant metastasis**	Yes	82	39 (47.6%)	43 (52.4%)	0.003*
	No	293	193 (65.9%)	100 (34.1%)	
**Chemotherapy**	Yes	103	58 (56.3%)	45 (43.7%)	0.173
	No	272	174 (64.0%)	98 (36.0%)	

*P<0.05 was considered significant

**Table 2:** Univariate and multivariate analysis of different prognostic parameters of ESCC patients (Cox regression).

Variates	Univariate analysis			Multivariate analysis	
	HR (95% CI)	*P* value		HR (95% CI)	*P* value
T stage (>T2 vs ≤T2)	1.797(1.326-2.435)	<0.001*		1.435(1.034-1.991)	0.031*
Lymphatic metastasis (Yes vs No)	1.775(1.332-2.367)	<0.001*			
pTNM stage (I-II vs III-IV)	1.847(1.387-2.459)	<0.001*		1.504(1.103-2.050)	0.010*
Differentiation (Well vs Moderate vs Poor)	1.282(1.061-1.550)	0.010*			
Postoperative metastasis (Yes vs No)	1.530(1.113-2.105)	0.009*			
Nerve invasion (Yes vs No)	1.617(1.123-2.329)	0.010*			
Chemotherapy (Yes vs No)	1.387(1.016-1.895)	0.039*			
MYADM expression (High vs Low)	1.901(1.427-2.534)	<0.001*		1.717(1.284-2.297)	<0.001*

*P<0.05 was considered significant

## Abbreviations

ESCC: esophageal squamous cell carcinoma
GEO: Gene Expression Omnibus
TCGA: The Cancer Genome Atlas
MYADM: myeloid-associated differentiation marker
EMT: epithelial to mesenchymal transition
EC: esophageal cancer
WGCNA: weighted gene coexpression network analysis
GO: gene ontology
KEGG: Kyoto Encyclopedia of Genes
PPI: protein-protein interaction
NSCLC: non-small-cell lung cancer
NCBI: National Center for Biotechnology Information
MAD: median absolute deviation
TOM: topological overlap matrix
DFI: disease-free interval
DSS: disease-specific survival
OS: overall survival
PFS: progression-free survival
TF: transcription factors
CTD: Comparative Toxicogenomics Database
TMA: tissue microarray
RIPA: radioimmunoprecipitation
SDS-PAGE: sodium dodecyl sulfate-polyacrylamide gel electrophoresis
OD: optical density
VRmRNAs: vascular remodeling related mRNAs
GTEx: Genotype-Tissue Expression
IHC: immunohistochemistry
TNM: the node and metastasis
TEC: tumor endothelial cells
