# Supplementary material for: Identification and Validation of MYADM as a Novel Prognostic Marker Related to EMT in ESCC
Source: J Cancer. 2024 Aug 19;15(16):5351–66. doi: 10.7150/jca.88767 (PMC11375559; doi:10.7150/jca.88767)
Supplement: Supplementary file 1 — Supplementary figures and tables. [file jcav15p5351s1.zip › supplementary legends.pdf]

Supplementary Figure 1. The validation of relationship between hub 4 genes-TFs-miRNAs regulatory network. (A) miRNA–hub 4 genes regulatory network: the red squares represent hub 4 genes, the purple dots represent miRNA, and the pink dots represent 4 key miRNA. (B) TF–hub 4 genes regulatory network: the red squares represent hub 4 genes, and the yellow dots represent transcription factors.

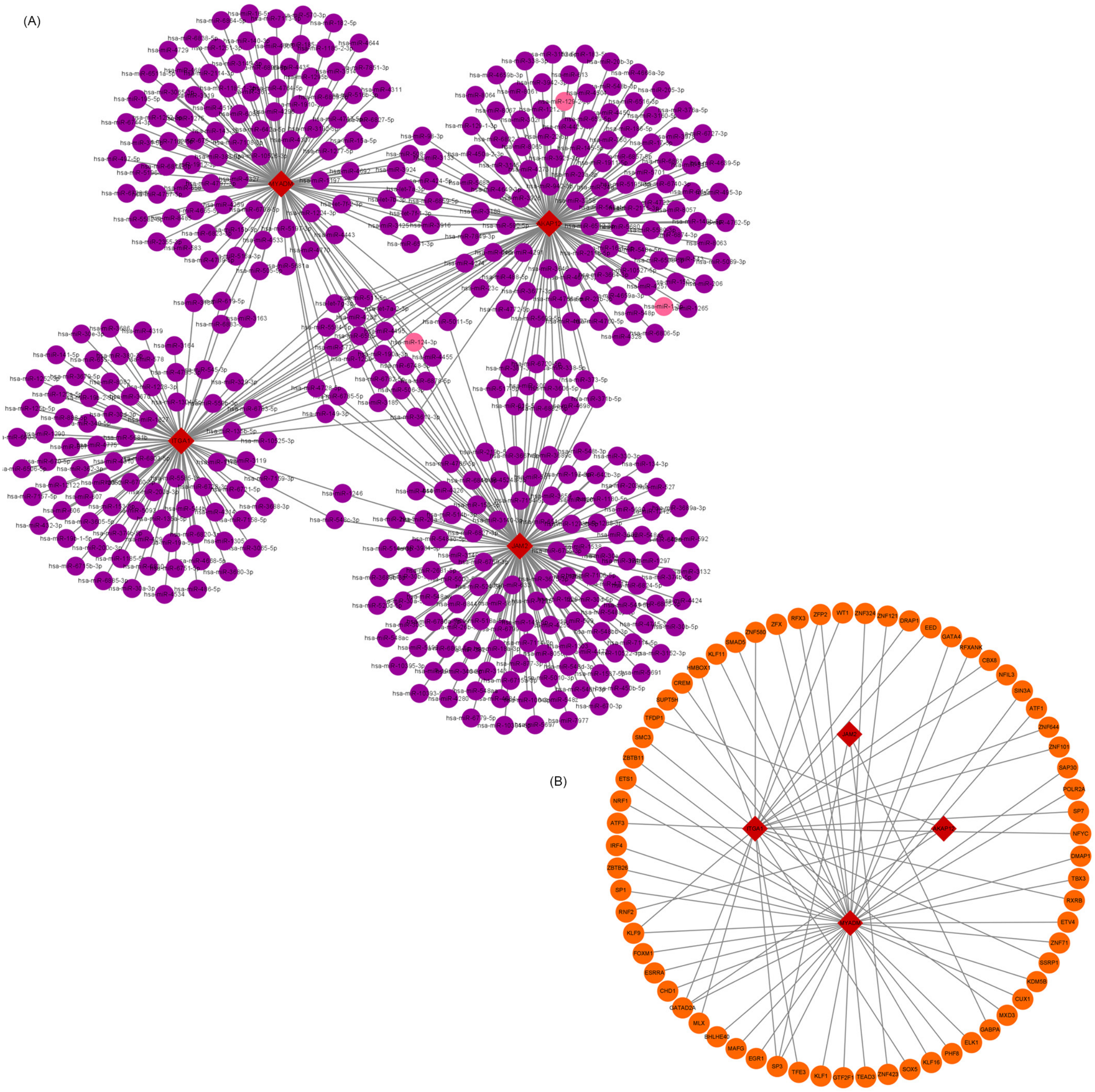

Supplementary Figure 2. Identification of the mRNA level of JAM2 (A), ITGA1 (B) and AKAP12 (C) based on the TCGA database in ESCC patients. The OS analysis of JAM2 (D), ITGA1 (E) and AKAP12 (F) based on TCGA. Summary of the JAM2 (G), ITGA1 (H) and AKAP12 (I) phosphorylation and ubiquitylation sites via PTM dataset.

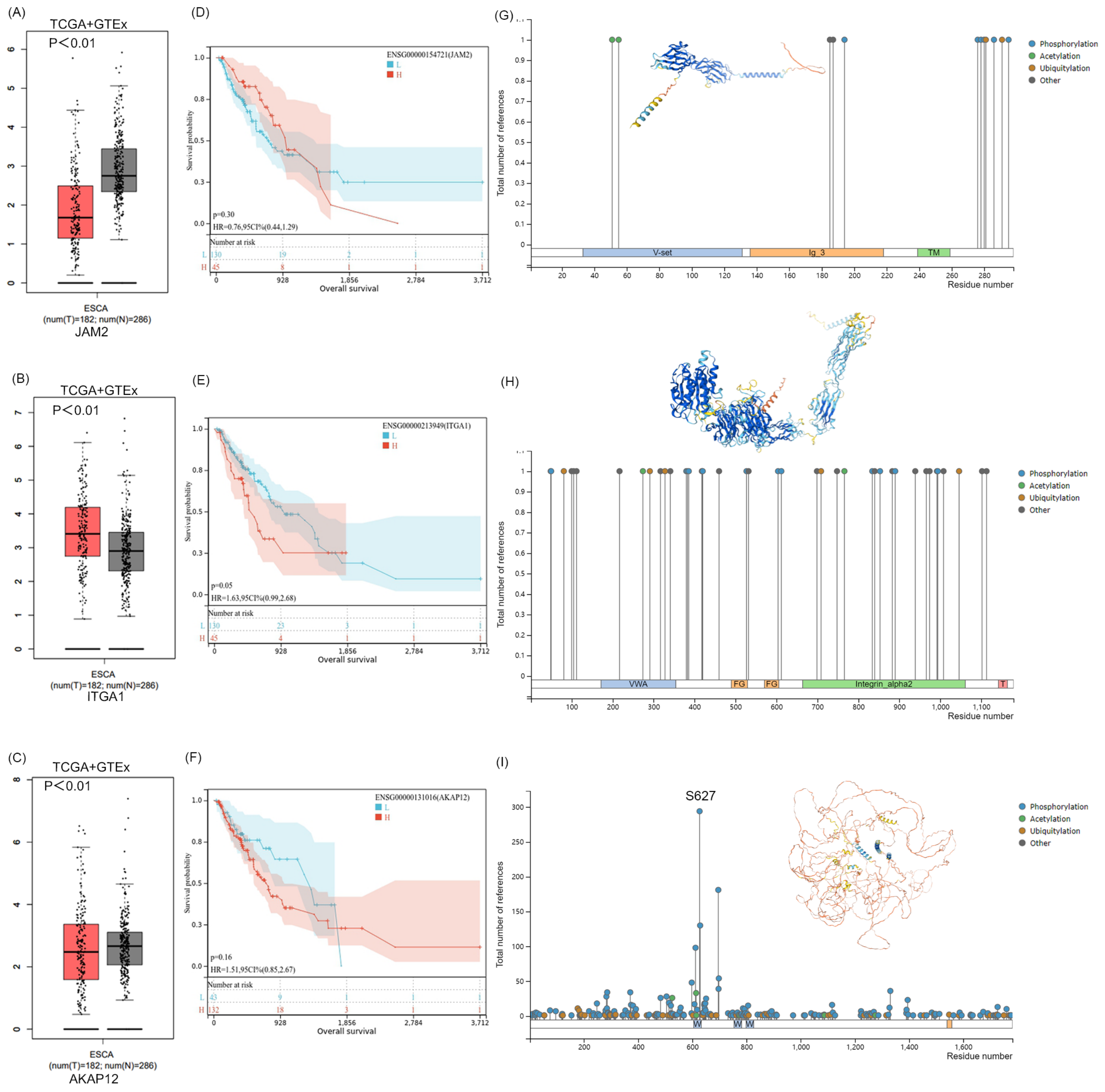

Supplementary Table 1 GeneCards-vascular remodeling.

Supplementary Table 2 GO functional enrichment analysis.

Supplementary Table 3: GSE45670clinical

Supplementary Table 4: GSE26886clinical

Supplementary Table 5: GSE75241clinical
