# Supplementary figures and images for: Identification and Validation of MYADM as a Novel Prognostic Marker Related to EMT in ESCC
Source: J Cancer. 2024 Aug 19;15(16):5351–66. doi: 10.7150/jca.88767 (PMC11375559; doi:10.7150/jca.88767)

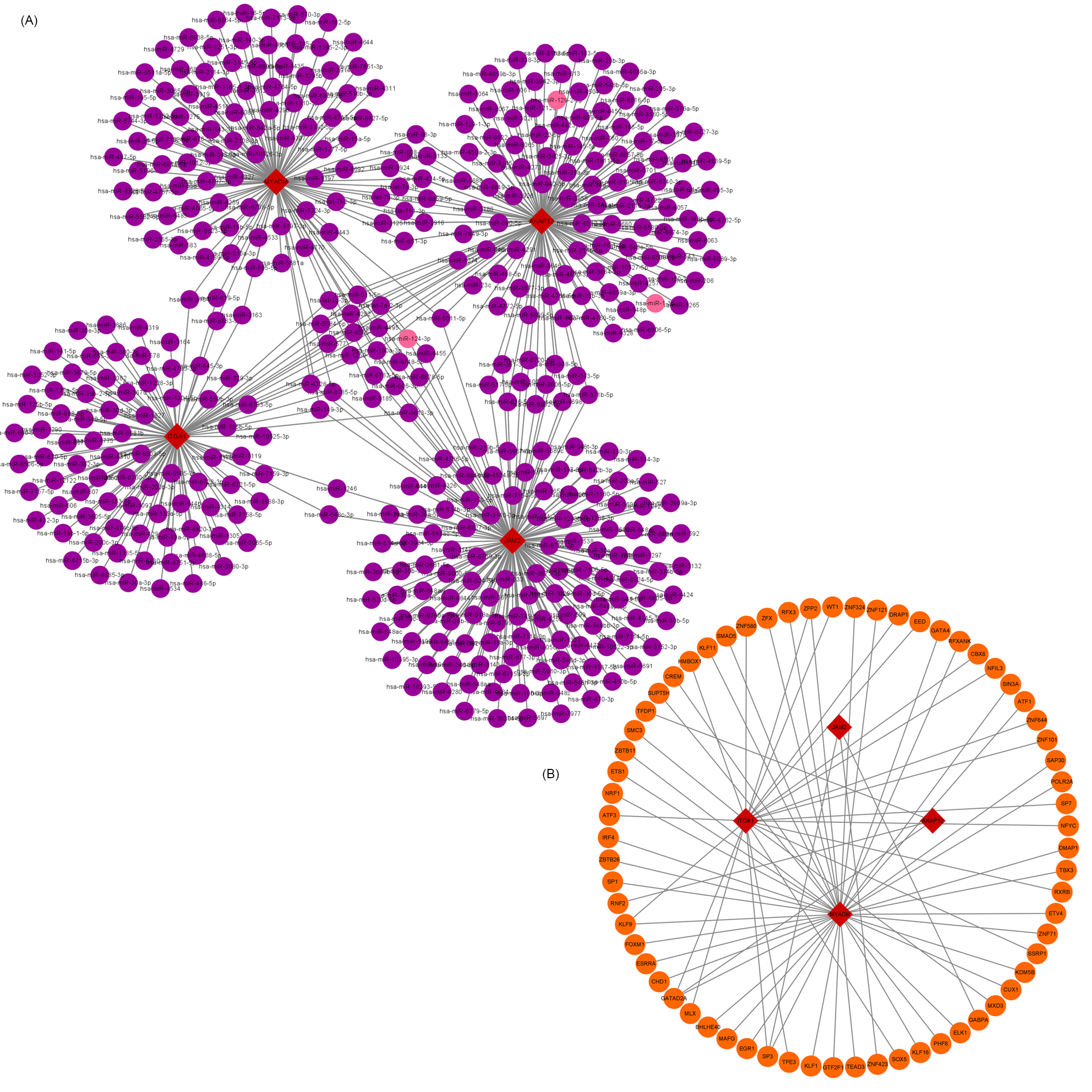

Supplement: Supplementary file 1 — Supplementary figures and tables. [file jcav15p5351s1.zip › SupplementaryFigure1.tif]

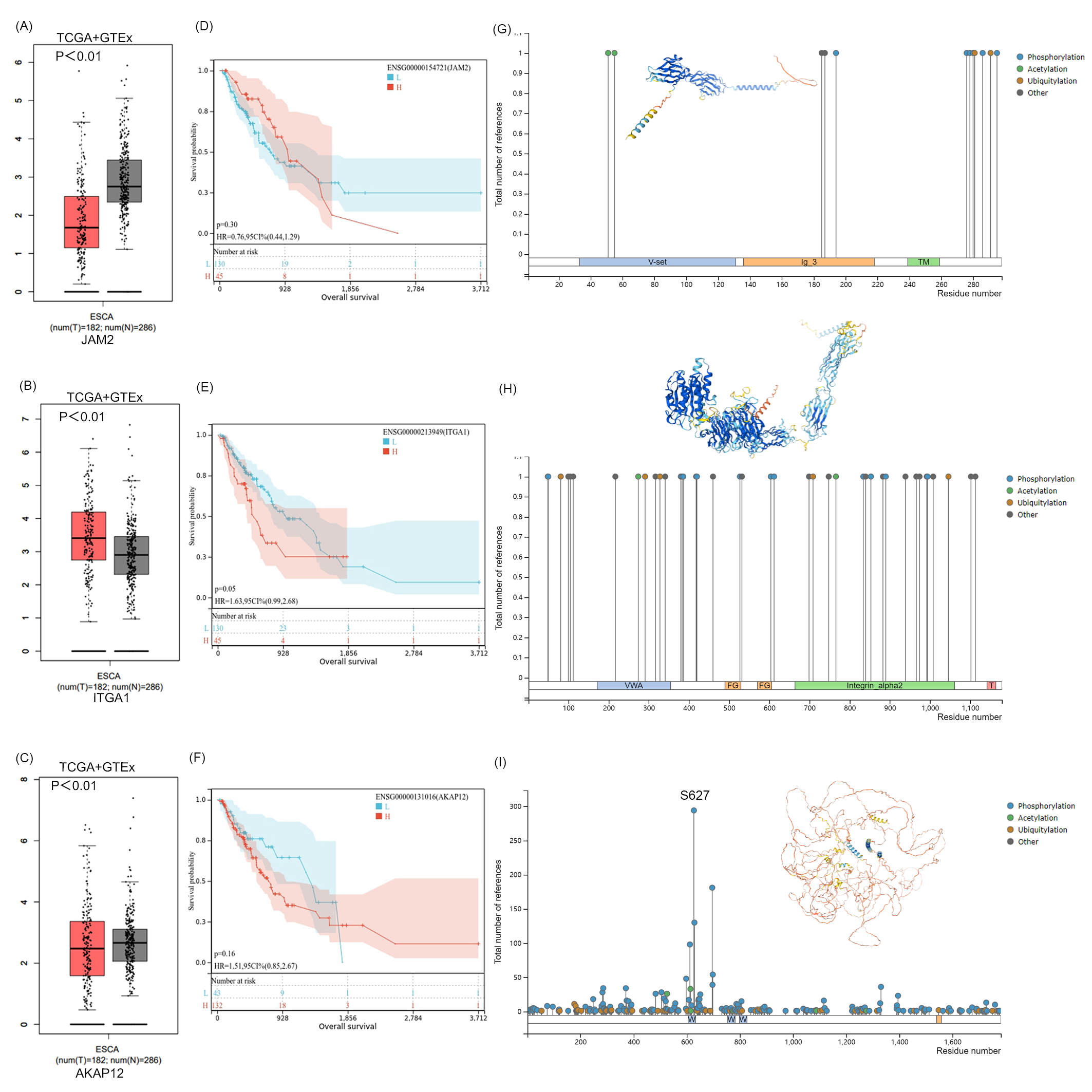

Supplement: Supplementary file 1 — Supplementary figures and tables. [file jcav15p5351s1.zip › SupplementaryFigure2.tif]
